# Knowledge of standard precautions and barriers to compliance among healthcare workers in the Lower Manya Krobo District, Ghana

**DOI:** 10.1186/s13104-017-2748-9

**Published:** 2017-08-30

**Authors:** Sandra Enyonam Akagbo, Priscillia Nortey, Mercy M. Ackumey

**Affiliations:** 1 Wisconsin International University College, Ghana, Box LG 751, Legon, Ghana; 2 School of Public Health, College of Health Sciences, University of Ghana, P. O. Box LG 13, Legon, Ghana

**Keywords:** Knowledge, Healthcare workers, Compliance, Standard precautions, Needle stick injuries, Barriers, Occupational health, Ghana

## Abstract

**Background:**

Implementing standard precautions (SP) has been a major challenge for health care workers (HCWs) especially those in developing countries thereby compromising their safety and increasing their exposure to blood-related pathogens. Compliance with safety precautions and occupational accidents among health workers are often unreported. The literature on knowledge and compliance to SP in Ghana is scanty. We report findings of a study that examined knowledge of SP, compliance and barriers to compliance with SP among HCWs in two health facilities in Ghana.

**Methods:**

This is a facility-based cross-sectional study involving 100 HCWs from two health facilities in the Lower Manya Krobo District of the Eastern region. Statistical analysis summarised data on socio-demographic characteristics of respondents, knowledge of SP and compliance and barriers to SP in frequencies and percentages.

**Results:**

Most respondents had been working as health staff for 0–5 years (65.0%). Generally, knowledge of the basic concepts of SP was low; only 37.0% of HCWs knew that SP includes hand washing before and after any direct contact with the patient, 39.0% knew about cough etiquettes and 40.0% knew about aseptic techniques which involve infection prevention strategies to minimise the risks of infection. Fifty percent of respondents always protect themselves against BBFs of patients. About a quarter of the respondents do not recap needles after use and 28.0% of respondents sometimes promptly wipe all blood spills. HCWs were of the opinion that wearing PPEs—such as gloves, aprons, gowns and goggles—might cause patients to panic sometimes (63.0%) and complying with SP sometimes interferes with the ability to provide care (38.0%). Sometimes, because of the demands of patient care, HCWs do not have enough time to comply with the rigours of SP (44.0%) and sometimes PPEs are not available.

**Conclusion:**

Education programmes on the benefits of SP should be organised frequently. The OHS national policy together with the application of the IPC training manual in all health care facilities must be enforced. Communities of practice should be established and sanctions and rewards should be introduced to limit negative behavior and reinforce positive attitudes as regards SP.

## Background

Guidelines to enhance the safety of Health Care Workers (HCWs) have been in existence since the late 1970s and early 1980s to help reduce the rate at which HCWs were exposed to blood, fluids, needles and other sharp objects [1]. This initiative was as a result of HCWs increased risk of exposure to blood-borne pathogens in the 1970s which led to the infection of many HCWs to hepatitis B virus (HBV) and human immunodeficiency virus (HIV) [1]. The content and labelling of the guidelines have changed over time. It was initially referred to as universal precautions or body substance isolation but now it is termed standard precautions (SP) [2].

The use of personal protective equipment (PPE) such as sterile surgical gloves and gowns, and sterile equipment, hygiene practices such as antiseptic hand washing, and safe instrument and waste disposal procedures as outlined in the SP guidelines can keep the HCWs safe from blood-borne infections [3–5]. However, implementing SP has been a major challenge for HCWs especially those in developing countries thereby compromising their safety and increasing their exposure to blood-related pathogens. This is due to shortage or lack of supplies, sub-optimal safety practices, poor training, poor awareness about the danger of unsafe infection control practices and limited organisational support for safe practice [5–9].

Studies suggest that non-compliance with safety precautions and occupational accidents among health workers are often unreported [10–13]. Furthermore, the literature on compliance and barriers to compliance of standard precautions in Ghana is scanty. In 2006, a study conducted among 50 medical personnel, ranging from medical students to consultants in one of the leading teaching hospitals in Ghana, the Korle-bu teaching hospital in Accra, suggested a gap in actual knowledge of SPs and practice [14]. A study conducted among 422 HCWs in the Greater Accra Region of Ghana revealed that HCWs who had adequate knowledge in the area of safety and health were more likely to comply with SPs [15]. Another study that examined knowledge and awareness levels of 108 nurses in the Tamale Metropolis of Ghana on exposure to the hepatitis B virus and the risk of infection ascertained that the majority of nurses (94.4%) considered themselves susceptible to HBV infection yet very few had adequate knowledge of post exposure prophylactic treatment against HBV [16].

Ghana’s Ministry of Health (MOH) which is mandated to set standards for the delivery of health care in the country, monitors and evaluates health service delivery by the Ghana health service (GHS), the teaching hospitals, other agencies, development partners and the private sector, provides a framework for the development and management of the human resources for health and makes proposals for the review and enactment of health legislation among others. By 2010, there were 52,258 individuals working formally in the health sector. These workers included those working in the public, private, Islamic missions, quasi-government health organisations and Christian health associations of Ghana [17].

Ghana has an occupational health and safety policy (OHS) which applies to all health institutions and administrative units within the health sector. The policy incorporates the health laws of the country and other international OHS protocols. This policy therefore provides the framework for the management of OHS to ensure the health and safety of workers of the health sector [18]. Additionally, there is an effective infection prevention and control (IPC) programme within the framework of the OHS policy. This is because, in the last two decades, health care-associated infections have been recognised as a significant heath problem that has compromised the quality of care and increased costs to patients, health care facilities, and the government. In 2005, a baseline assessment of IPC in major health facilities provided discouraging evidence of compliance with IPC guidelines by health personnel. Health personnel exhibited poor knowledge about disinfection of hands, sterilisation of equipment, cleaning, waste management, and other aseptic procedures in health-care settings. To this end, the first edition of the national IPC policy and guidelines document to streamline safety measures was developed in 2003 and reviewed in 2009 to incorporate emerging infections [19].

Against this background, we report findings of a study that examined knowledge of SP, compliance and barriers to compliance with SP among HCWs in two health facilities in Ghana. Identifying factors that influence poor knowledge of SP and non adherence to SP is important to design public health programmes that offer pragmatic strategies to ensure the adherence of SP across all health facilities for the protection of HCWs.

## Methods

### Study design and study area

This is a facility-based cross-sectional study involving 100 HCWs from two health facilities—the Akuse Government hospital and the St. Martin de Porres hospital (St. Martin’s, for short)—in the Lower Manya Krobo district of the Eastern region. The Eastern region was purposively selected because it had the highest HIV prevalence of 3.6% in the country in 2012 and maintained the lead in HIV prevalence until 2015 [20]. Additionally, it has a high percentage of sex workers and men who have sex with men (MSM) [21].

The Akuse Government hospital and the St. Martin de Porres hospitals are the two major referral facilities for HIV related cases in the district. Both hospitals offer a full range of HIV/AIDS-related services such as voluntary counselling and testing (VCT) and prevention of mother to child transmission (PMTCT) and were the first PMTCT pilot sites in Ghana. The St. Martin’s hospital also undertakes home based care services for persons living with HIV and AIDS (PLWHA’s). Data was collected from May to June, 2013.

### Study population

HCWs are “all people engaged in actions whose primary intent is to enhance health” [22]. These include nurses, physicians, pharmacists, technicians, morticians, dentists, medical students and first aid providers or volunteers. For the purpose of this study, HCWs comprised of nurses, ward aides or ward orderlies, laboratory technicians and midwives since they often come into contact with blood and body fluids (BBFs) during treatment especially in emergencies. Medical doctors were classified in another group and were not considered to come into frequent contact with patients as compared to nurses, ward aides or ward orderlies, laboratory technicians and midwives. The total number of HCWs for both facilities was 172.

### Sample size and sampling

An initial sample size of 77 was calculated using the formula for populations less than 10,000 persons.

$${\text{nf}} = \frac{\text{n}}{{1 + \frac{{({\text{n}})}}{{({\text{N}})}}}}$$

In this formula, nf is the desired sample size when the population is less than 10,000; n = 138 and it is the desired sample size when the population is more than 10,000 (this was computed using a Cochran formula, for 10% of the study population, which is ${\text{n}} = {\text{z}}^{ 2}\,{{\text{pq}}\mathord{/ {\vphantom{{\text{pq}}{{\text{d}}^{2}}}}\kern-0pt} {{\text{d}}^{2}}},$ where z is estimated at 1.96 for a 95% confidence level, p is 10% of the estimated study population [10,000, expressed as a decimal (0.1)] and d is the level of acceptable error estimated at 5% [23]. The sample size for the population was estimated at 77. However, after adjusting for low response rates because HCWs are very busy and considering the need to increase the sample size for a meaningful statistical analysis, the research team agreed to interview 100 HCWs.

The sample of 100 HCWs was split between the 2 hospitals in the ratio 52:48 for the Akuse government hospital and the St. Martin’s hospital respectively. The 100 HCWs comprised midwives, nurses, laboratory technicians and ward orderlies. The selection of these categories of health workers was based on their frequent interaction with patients and handling of hospital treatment equipment which often exposed them to potentially infectious BBFs.

Using a quota sampling technique based on the proportion of HCWs in each category, 7 midwives, 30 nurses, 5 laboratory technicians, and 10 ward orderlies were sampled from the Akuse government hospital while 11 midwives, 25 nurses, and 22 ward orderlies were sampled from St. Martin’s hospital. It was intended to sample laboratory technicians from the St. Martin’s hospital but this was not possible because they had other work-related commitments. In total, 18 midwives, 55 nurses, 5 lab technicians and 22 ward orderlies participated in the study, (Table 1). A list of all HCWs in each hospital was obtained. A simple random sampling technique was used to select respondents. Where a selected individual was unavailable or declined to participate, a proxy HCW in the same category on the list was chosen.

**Table 1:** Distribution of participants from Akuse and St. Martin hospitals

HCWs	Akuse government hospital		St. Martin’s hospital		Total	
	Freq.	Perc.	Freq.	Perc.	Freq. (N = 100)	Perc.
Midwives	7	7.0	11	11.0	18	18.0
Nurses	30	30.0	25	25.0	55	55.0
Laboratory technicians	5	5.0	0	0	5	5.0
Ward orderlies	10	10.0	12	12.0	22	22.0

*Freq*. frequency, *Perc*. percent

### Data collection tool

A structured questionnaire was used to obtain data from respondents. Since there are few studies on knowledge of SPs in the local context, a review of existing studies was conducted to provide a background for the study and provide plausible questions on the research topic. Two main studies [5, 24] guided the formulation of the questionnaire for this study. Questionnaires elicited information on demographic information of respondents, knowledge of SP, compliance with SP and barriers to compliance, work related injuries and risk perception of HIV among health workers. However this paper presents findings of knowledge of SP and barriers to compliance among HCWs.

### Pre-testing and data collection

The questionnaire was pre-tested by the first author at the University of Ghana hospital in the first and second week of May 2013. Two members of the research team met for a day towards the end of the third week of May to review the results of the pre-test, to check for clarity of questions and to eliminate repetitive and ambiguous questions. Similar to the St. Martin and Akuse government hospitals, the University of Ghana hospital has trained physicians and dedicated nursing and pharmacy staff who have been fully engaged in developing ART programmes and models of care. The hospital offers PMTCT, voluntary counseling and testing (VCT) and family planning services. It also has an out-patient and in-patient department, a theatre, an antenatal clinic, a dental clinic, an eye clinic, a functioning laboratory, a pharmacy, a laundry and mortuary. Data was collected for 4 weeks within the last 2 weeks of May and June, 2013.

### Data management and analysis

Data was double—entered in a Microsoft excel spreadsheet to reduce data entry errors, later exported into STATA 12 (StataCorp LP, College Station, TX, USA) and analysed using STATA 12. First, the Cronbach’s alpha, a statistical test that measures the internal consistency or reliability of Likert scale questions was performed on questions related to compliance to SP and barriers to compliance of SP; the test produced alpha scores of 0.72 and 0.80 respectively. The acceptable values of alpha range from 0.7 to 0.95 [25, 26]. Statistical analysis summarised data on socio-demographic characteristics of respondents, knowledge of SP and, compliance and barriers to SP in frequencies and percentages.

## Results

### Background characteristics of respondents

The majority of respondents were female (73.0%). With respect to categories of health care providers, 55.0% were general nurses, 22.0% were ward orderlies 18.0% were midwives and 5.0% were laboratory technicians. Most respondents had been working as health staff for 0–5 years (65.0%), (Table 2).

**Table 2:** Background characteristics of respondents

Variables	Frequency (N = 100)	Percentage (%)
Sex		
Males	27	27.0
Females	73	73.0
Area of practice		
General nurse	55	55.0
Midwives	18	18.0
Laboratory technician	5	5.0
Ward orderlies	22	22.0
Highest level of education		
Senior high school/vocational school	23	23.0
Tertiary	77	77.0
Years of practice		
0–5	65	65.0
6–11	23	23.0
12–17	5	5.0
18–23	0	0
24–29	1	1.0
30+	6	6.0

### Knowledge of standard precautions among HCWs

Knowledge of SP focused on respondents understanding of practices adopted to prevent infection from BBFs. Generally, knowledge of the basic concepts of SP was low among HCWs. Only 37.0% of HCWs knew that SP includes hand washing before and after any direct contact with the patient, 39.0% knew about cough etiquettes and 40.0% knew about aseptic techniques which involve infection prevention strategies to minimise the risks of infection (Table 3). Respondents mentioned NSIs (67.0%), inhalation (64.0%) and talking and touching patients as potential ways of occupational exposure (64.0%). Knowledge of hand washing practices and the use of PPEs was generally poor. About half of the HCWs were knowledgeable about important factors to consider when deciding when to use PPEs. At least 90.0% of respondents stated that SP should be applied for protection against blood (92.0%), vaginal fluids (91.0%), blood tinged body fluids (91.0%) and saliva in dental procedures (91.0%), (Table 3).

**Table 3:** Knowledge of standard precautions among HCWs

Knowledge of standard precautions among HCWs	Freq.	Perc.
*The concept of standard precautions includes*		
Hand washing before and after any direct contact with patient	37	37.0
Consideration of the potential for transmission of infectious agents to patients	38	38.0
Cough etiquette such as directing patients/relatives with symptoms of a respiratory infection to cover their mouths/noses when coughing or sneezing	39	39.0
Safe injection practices such as aseptic techniques	40	40.0
*Potential ways of occupational exposure*		
Needle stick/sharp injury	67	67.0
Splash on the eye	65	65.0
Inhalation	64	64.0
Talking to patients	64	64.0
Touching patients	64	64.0
*According to the SP*, *hand washing is performed*		
Before any direct contact with patients	55	55.0
Between patients’ contact	54	54.0
Immediately after removing gloves	53	53.0
After touching body fluids such as blood, excretions and sweat	58	58.0
*For which of these conditions should SP be followed*		
To all hospitalised patients	93	93.0
When the healthcare worker has a known or suspected infection	91	91.0
When the patient has a known or suspected infection	90	90.0
At the discretion of the healthcare worker.	90	90.0
*Body fluids that require SP*		
Blood	92	92.0
Vaginal fluids	91	91.0
Blood tinged body fluids	91	91.0
Saliva in dental procedures	91	91.0
*Important factors in deciding when to use PPEs such as goggles*, *mask*, *gloves*, *gowns and apron*		
HIV/AIDS	52	52.0
Hepatitis B virus (HBV) infection	51	51.0
Signs and symptoms of infection	53	53.0
*Post exposure prophylaxis* (*PEP*) *for HIV*		
HIV counseling and testing is done immediately after the exposure	49	49.0
PEP is given only to HIV negative test result	49	49.0
Two or three antiretroviral drugs are given immediately after the exposure but within 72 h	90	90.0
The antiretroviral drug is taken for 4 weeks	64	64.0

Results of multiple choice questions are reported in this table so N exceeds 100 for thematic areas in italic text

*Freq*. frequency, *Perc*. percent, *SP* standard precautions, *PPE* personal protective equipment

### Compliance with standard precautions

Table 4 reports respondents’ compliance with SP. Half of the respondents always protect themselves against BBFs (50.0%). About a quarter of the respondents do not recap needles after use (25.0%). Twenty-eight (28.0%) percent of respondents sometimes promptly wipe all blood spills while 61.0% of respondents always wipe blood spills. Surprisingly, only 61.0% of respondents wear gloves, the basic protective equipment. As regards training, 40.0% of respondents mentioned that supervisors encourage training in SP and 48% of HCWs had regular training in SP (Table 4).

**Table 4:** Compliance with standard precautions

Variables	Degree of compliance									
	Never		Rarely		Sometimes		Often		Always	
	*F*	%	*F*	%	*F*	%	*F*	%	*F*	%
Protection against BBFs of all patients	0	0.0	1	1.0	25	25.0	24	24.0	50	50.0
Puts used needles into sharp container	1	1.0	1	1.0	13	0.0	30	30.0	55	55.0
Wears gloves	0	0.0	1	1.0	10	10.0	28	28.0	61	61.0
Wash hands after removing gloves	0	0.0	1	1.0	21	21.0	29	29.0	49	49.0
Wears waterproof apron	2	2.0	11	11.0	35	35.0	26	26.0	26	26.0
Wears eye protection	9	9.0	25	25.0	37	37.0	12	12.0	17	17.0
Does not recap needles	25	25.0	18	18.0	17	17.0	6	6.0	34	34.0
Promptly wipes all blood spills	1	1.0	2	2.0	8	8.0	28	28.0	61	61.0
Covers broken skin	2	2.0	4	4.0	8	8.0	28	28.0	61	61.0
Reports needle-stick injury	8	8.0	7	7.0	27	27.0	25	25.0	33	33.0
Supervisors encourage training	6	6.0	6	6.0	40	40.0	19	19.0	29	29.0
Staff have training in SP	3	3.0	0	0	23	23.0	26	26.0	48	48.0

Frequencies for this table are based on the sample size of 100 respondents

*F* frequency, *%* percent, *BBFs* blood and body fluids

### Barriers to compliance of safety precautions

Many HCWs mentioned that sometimes PPEs are not available (74.0%). Half of the respondents mentioned that complying with SP in emergency situations sometimes places the patients at risk of adverse situations or death (50.0%). Sometimes, because of the demands of patient care, HCWs do not have enough time to comply with the rigours of SP (44.0%), (Table 5). HCWs were of the opinion that wearing PPEs—such as gloves, aprons, gowns, goggles and placing used needles in ‘sharps’ containers—might cause patients to panic sometimes (63.0%) and sometimes complying with SP interferes with the ability to provide care (38.0%). Because of the unanticipated exposure to infection, 39% of respondents sometimes fail to comply with SP, (Table 5).

**Table 5:** Barriers to compliance of standard precautions

Barriers to compliance of standard precautions	Never		Rarely		Sometimes		Often		Always	
	F	%	F	%	F	%	F	%	F	%
Compliance during emergency puts patients at risk	17	17.0	8	8.0	50	50	25	25.0	0	0
Complying with SP interferes with the ability to provide care	26	26.0	10	10.0	38	38.0	19	19.0	7	7.0
Exposure to infection is unanticipated	17	17.0	19	19.0	39	39.0	15	15.0	10	10.0
Patient care demands does not allow ample time to comply with SP	9	9.0	12	12.0	44	44.0	26	26.0	9	9.0
Unavailability of equipment	10	10.0	3	3.0	74	74.0	7	7.0	6	6.0
Patients do not pose a risk	25	25.0	12	12.0	38	38.0	17	17.0	8	8.0
Protective gear is uncomfortable	25	25.0	12	12.0	42	42.0	16	16.0	5	5.0
Ineffective equipment	10	10.0	12	12.0	60	60.0	12	12.0	6	6.0
Wearing protective equipment might cause fear in patients	19	19.0	11	11.0	63	63.0	4	4.0	3	3.0
PPE is not conveniently located to enable use	17	17.0	10	10.0	53	53.0	17	17.0	3	3.0
Practice of SP is time consuming	30	30.0	11	11.0	45	45.0	9	9.0	5	5.0

Frequencies for this table are based on the sample size of 100 respondents

*F* frequency, *%* percent, *SP* standard precautions, *PPE* personal protective equipment

## Discussions

Study findings indicate that knowledge of the basic concepts of SP was low among HCWs. HCWs demonstrated limited knowledge on potential pathways of occupational exposure, hand washing routines which is a basic standard precaution and the use of PPEs and post exposure prophylaxis. Our study findings are similar to other study findings where knowledge of basic concepts of SP was low [3, 27–29].

HCWs for this study were recruited from two major hospitals that offer HIV services. These hospitals register the highest HIV infections in the country and were the first ART and PMTCT sites in the country. It is therefore worrying that HCWs in these facilities have limited knowledge of SP. This suggests the need for periodic education and training programmes to improve knowledge of SP and awareness of occupational exposure to HIV and its management to prevent infection of HCWs and cross infection of patients. These training programmes must emphasise the pathways to, and the likelihood of nosocomial infections. Regular drills on SP should be organised on monthly basis to reinforce education. Additionally, distribution of flyers or leaflets on SP, placing posters in vantage areas in the health centre and videos are some measures that could be adopted to reinforce education on SP.

### Compliance with standard precautions

Compliance with SP is the hall mark of health care practice and is effective in the prevention of BBFs. Study findings showed that many HCWs were non compliant with SP: Only half of the study respondents protect themselves against BBFs. Very few respondents always wear eye protection and water proof aprons and about a third of respondents recap needles after use and always report NSIs. NSIs often account for most of the occupational injuries and exposure to harmful bacteria and infection among health workers [13, 30, 31] and therefore have to be reported promptly for emergency treatment or the use of PEPs. Other studies have reported partial compliance with SP or sub-optimal practices in Ghana [32] Nigeria [5, 27, 28], Tanzania [29], Uganda [33], Ethiopia [7, 13, 34], India [1], Italy [35] and Cyprus [36]. There is enough research evidence to suggest that adequate knowledge and education through training programmes about SP modifies HCWs behaviour and attitudes and therefore influences compliance [35, 37, 38]. It behooves all health facilities to set up systems that enforce compliance with SP for the sanity of the health environment. This system should factor in a well coordinated supervisory and reporting system, where accidental splashes and spills of BBFs, contagion, NSIs and any activity that amounts to non compliance to SP is noted and reported. All health staff should be conscientised about SP and ensure that colleagues adhere strictly to SP, without compromise. Furthermore, all health centres should be encouraged to establish communities of practice (CoP) where health staff could learn from colleagues and share experiences about adhering to SP.

### Barriers to compliance of standard precautions

Many respondents (74.0%) reported the unavailability of PPEs as a barrier to compliance of SP. The absence or insufficiency of basic protective equipment such as masks, gloves and goggles have been reported as barriers to compliance with SP in many studies [36, 39–41]. One study in India reported the absence of PPEs, especially during emergencies [42], studies in Nigeria reported the absence or inadequacy of PPEs [28, 43, 44] and a study in China mentioned inadequate provision of eye shields, protective masks, quarantine clothes and shoe covers as barriers to compliance [38]. In Malaysia, the unavailability of gloves at emergency sites was cited as a reason for irregular glove use [40], a study among paediatric care units in Egypt cited inadequate protective equipment as one of the reasons for non-compliance with SP [37] and in Ethiopia, lack of supplies of PPEs was mentioned as a hindrance to compliance [13].

Another barrier to adhering to SP in this study was the discomfort of PPEs when worn. This study finding is consistent with findings from other studies: HCWs in India indicated that wearing PPEs is uncomfortable [42]. HCWs in Brazil mentioned that PPEs are uncomfortable [45] and make HCWs hot and uncomfortable, given that Brazil is a tropical country. Furthermore, HCWs in Sierra Leone considered PPEs uncomfortable as it makes them hot and induces sweating and itching although they acknowledged the benefits of PPEs for the prevention of infections [46]. Nurses working in 2 government hospitals in Cyprus complained that using gloves decreases dexterity when drawing blood [36]. HCWs do not only have to wear PPEs for protection against BBFs such as HIV and HBV but also for protection against deadly viruses such as Ebola. Infection prevention controls in the national IPC manual must be adhered to as a matter of policy. Secondly, extensive health education programmes about the benefits and proper use of PPEs must be heightened during training programmes of HCWs. Furthermore, the proper use and removal of PPEs as illustrated in the IPC manual must be adhered to strictly.

Another study finding was that HCWs sometimes considered compliance during emergencies as risky to patients (50.0%) and the practice of SP as sometimes time consuming (45.0%). This finding is consistent with findings from earlier studies [1, 36, 47, 48]. Some authors argue that because of the high work loads of HCWs, particularly in developing countries, and time limitations, wearing different protective wear and the rigours of hand washing in between handling patients is considered burdensome, interfering with their duties and placing patients at the risk of escalating sickness [9, 49–53]. The wearing of double gloves over single gloves is recommended as it provides better protection from NSIs and serves as a stronger barrier against BBFs [54, 55]. However, studies show that HCWs argue that double-gloving interferes with their operations as it induces heat, impairs dexterity and limits sensation [54, 56, 57]. As a matter of public health policy, health care facilities must ensure that hand hygiene and proper use of gloves must be optimised to protect the patient and HCW and indirectly minimise treatment costs of cross infections as a result of suboptimal hand hygiene care. Alcohol-based hand rubs have been shown to improve health care workers’ compliance with hand hygiene practices [58]. The provision and use of alcohol-based handrubs before and after handling patients and BBFs should be enforced.

Another reason for non-compliance in our study was the notion that wearing of protective clothing might instill fear in patients. Our study findings corroborate other study findings. In a study in Cyprus, HCWs mentioned that patients may experience anxiety, distress or sadness when nurses wear protective wear such as masks, gowns and gloves [36]. In China, HCWs mentioned that patients are uncomfortable with protective wear [38] and in a study conducted in Brazil, HCWs indicated that the use of protective wear may cause psychological distress among patients [56]. To encourage the wearing of PPEs, the work environment must be improved especially for developing countries. Suggested improvements are the inclusion of cooling systems such as fans and air conditioners to make wearing of PPEs more comfortable. Patients must be made aware that PPEs also protect them from infection; this may minimise psychological distress. As much as possible, all health workers should be involved in decisions governing SP. Supervisors must reinforce the need to wear PPEs correctly and regularly. Sanctions for noncompliance and rewards for compliance of SP should be instituted to promote compliance to SP. Reprimanding HCWs for non-compliance of SP was found to be effective [52].

### Study limitations

Since this study relied on recall of past behaviour the information may be prone to recall bias. Information obtained from HCWs was not validated through direct observation. We interviewed respondents who were available to be interviewed and this accounts for the low numbers of laboratory technicians that were included in the study. Nevertheless, study findings provide insights into compliance to SP and reasons for non-compliance among HCWs in two important health facilities in Ghana and serve as a basis for further studies.

Future research on SP and occupational hazards among HCWs in Ghana should examine the role of incentives and sanctions to enforce SP, workloads and occupational exposures, health worker safety culture and patients knowledge and perceptions of the use of PPEs.

## Conclusion

Study findings suggest that knowledge of the basic concepts of SP was low among HCWs. HCWs did not wear PPEs regularly. The unavailability of PPEs, discomfort of wearing PPEs and the notion that adherence to SPs was time consuming were some barriers to compliance. Education programmes on the benefits of SP should be organised frequently. The OHS national policy together with the application of the IPC training manual in all health care facilities must be enforced. Communities of practice should be established and sanctions and rewards should be introduced to limit negative behavior and reinforce positive attitudes as regards SP.

## Abbreviations

AIDS: acquired immune deficiency syndrome
ART: antiretroviral therapy
BBF: blood and body fluids
CoP: community of practice
GHS: Ghana health service
HBV: hepatitis B virus (HBV)
HCW: health care worker
HIV: human immunodeficiency virus
IPC: infection prevention and control
MOH: Ministry of Health
NSI: needle stick injury
OHS: occupational health safety
PEP: post exposure prophylaxis
PLWHAs: persons living with HIV and AIDS
PPE: personal protective equipment
PMTCT: prevention of mother-to-child transmission
SP: standard precautions
VCT: voluntary counselling and testing
